# Dr. James Annesley on the Effects of Calomel in Indian Diseases, and on the Mucous Membrane of the Stomach and Bowels

**Published:** 1826-04

**Authors:** 


1. *Practical Observations on the Effects of Calomel on the Mucous Surface and Secretions of the Alimentary Canal; and on the Use of this Remedy in Disease, more particularly in the Diseases of India.* By JAMES ANNESLEY, Esq. Madras Medical Establishment. Octavo. London, 1825.

2. *An Essay on Mercury in Syphilis and other Diseases.* By S. A. CARTWRIGHT, M.D. of America.

OF late years, certain prejudices and erroneous opinions, respecting the *doses* of medicines, have begun to give way. The Italian physicians have let in some light on these matters, and the medical men of the Continent, generally, are taking advantage of it. It is now some 20 years, since a few medical men, employed in the naval service in India, ventured, in certain dangerous cases of dysentery and hepatitis, to deviate from the ordinary course of procedure, and exhibit the submuriate of quicksilver in doses which alarmed the prejudices of the *routinists*, and afforded a fine opportunity for every old woman and young cub in the profession, to hold forth on the injurious tendency of the practice. It was in vain that the gentlemen alluded to appealed to facts, and offered to submit to the test of experiment. No such indulgence was to be granted. It was known that three or four grains of calomel would produce three or four motions, generally with griping pains, and it was not merely inferred from thence, but considered as a mathematical demonstration, that five or six times the dose must necessarily produce five or six times the effects abovementioned—that is, that a scruple of calomel would be followed by such hypercatharsis and pain, as would, most likely, destroy the unhappy patient who had taken it. The few who tried the experiment were not believed when they published the result, and the consequence was that many who, in the end, became convinced of the errors of the scholastic dogmas, kept their heresies to themselves, in order that they might not be exposed to the persecutions of the ignorant and illiberal. Evidence, however, has accumulated on this point, and since the late experiments of the Italian, and other Continental physicians, on antimony and different active articles of the Materia Medica, scepticism has abated, and Intolerance has somewhat changed her note. The portion of Mr. Annesley's work which we are now about to review, contains many important remarks on the diseases of India, and on the influence of calomel in their treatment—and it is probable that the *modification* which he has introduced into practice may turn out an *improvement*, which is more than falls to the lot of every peculiarity in therapeutics.

We shewed, some time ago, (*Med. Chir. Rev. for June*, 1823, *p* 222,) that the practice of giving the submuriate of mercury in the stigmatised scruple doses, was no new practice in the fevers and dysenteries of tropical climates—since the plan was adopted, more than 60 years ago, by Dr. Smith, Dr. Wright, and others, in the West Indies. We also quoted some of the earlier practitioners of modern times, who were in the habit of exhibiting even larger doses of the medicine, as a purgative or cholagogue, in this country and on the Continent. Mr. Annesley has collected still more numerous testimonies than we thought it necessary to adduce in corroboration of the safety of the practice now advocated. We shall put some of these again on record. Horstius (Op. Omnia, vol. ii. p. 480) states, that mercurius dulcificatus, or calomel, may be given in doses of one scruple, or half a drachm, "ad viscidos humores magis attenuandos;" and D. Sylvius recommends it, in the same doses, as a purgative. Wepfer prescribed it in scruple doses, combined with other purgatives, in affections of the head; and so did Dr. Friend, in Emansio and Obstructio Mensium. Scheoder, Junker, Geoffroy, and many others, might be quoted, but enough has been said to shew that the same fears were not entertained of full doses of calomel in those times, that now ring so often in our ears.

Mr. Annesley, after some judicious reflexions on the difficulty of eradicating the prejudices of early education, and on the proneness of our nature to take for granted what has been stated by others rather than investigate for ourselves, proceeds thus:—

"Influenced, in some degree, by a similar partiality of judgment and indolence in research, which I here condemn, I was in the habit of administering calomel only in moderate doses, until I perused the valuable work of Dr. Johnson, after which I began, in my hospital at Trichinopoly, to exhibit it in doses of one scruple each, to a patient in the advanced stage of dysentery: its action in this instance, was so strikingly useful in procuring ease and comfort to the patient, that, although the case was not successful, I determined to give it a trial at the commencement of those acute diseases which we find most distressing and destructive in India, namely, in dysentery, hepatitis, and fever,—diseases which, in general, commence with great excitement, and excessive irritability of stomach. I accordingly adopted this practice, and have followed it for upwards of eight years, and in no instance have I had reason to be dissatisfied with its effects.

"Having been even at that time prepossessed in favour of large doses of calomel, it was not a difficult matter to make me a convert to the practice; but I adopted it with very different views from those with which it was then recommended, and modified it accordingly, as will be seen in the sequel: nevertheless, so great were the prejudices that existed against this practice, even amongst men of professional eminence and reputation, that I have often doubted my own judgment in suggesting to those gentlemen who were placed under me, on their first arrival in India, the propriety of administering calomel in larger doses than are commonly thought necessary, although the result of my own experience was so decidedly in favour of the practice; and I have sometimes felt great difficulty in meeting, and successfully resisting, the various objections which have been made to it. I consequently did not press its use, but gave the confident assurance that calomel could be used in large doses with perfect safety; and established the fact by shewing its effects when so administered by me. A very short time convinced them of its advantages, and the practice became general from conviction, and not from persuasion.

"It is generally believed, and probably it may be true, that many constitutions in India are ruined by the use of calomel; but I am disposed to consider this to be the consequence of continuing it in small doses, long after the necessity for using it ceases." 385.

Mr. Annesley observes, that it is that class of practitioners who decry the use of large doses of calomel "*who really give infinitely larger quantities*," although in small and repeated doses. This is strictly true, and we apprehend that, in those urgent cases, where large doses are necessary, the same objection will, in a degree, apply to Mr. Annesley's own plan—that of a single dose daily. We are not among those who would wantonly, and upon trifling occasions, bring the system under the influence of mercury; but knowing, as we do, that nothing short of this will save life, under certain conditions of disease, we should, notwithstanding Mr. Annesley's authority to the contrary, prefer the double or even treble exhibition of the medicine in the 24 hours, to the single evening dose, where life depended on the result. But more of this hereafter. We agree with our author, or rather he repeats what we many years ago asserted, that:—

"Small doses of calomel, from two, three, to four and six grains, will purge, and keep up a considerable degree of irritation in the stomach and bowels, when twenty grains will not; but, on the contrary, will allay the irritation of both, when it results from inflammation of their mucous surfaces. Thus, calomel, in large doses, appears to act as a sedative, as will be proved by the experiments I am about to adduce." 387.

The discrepancies of opinion respecting calomel in the diseases of India, induced our author to institute some experiments upon dogs, with a view of elucidating the subject; and the results were so satisfactory, he thinks, as to warrant their publication for the information of the profession. We shall endeavour to convey some idea of these experiments to our readers.

*Exper.* On the 1st December, 1823, the doses of ʒj., ʒij., and ʒiij. of calomel were given to three healthy dogs. After taking the calomel they were kept in a room and narrowly watched for 24 hours.

"The dog who took ʒj. did not appear to feel any kind of sickness till night, when he vomited a little; he was lively the whole time, and ate his food well; had been purged two or three times; dejections of a very dark grey colour.

"The dog who took ʒij. was likewise lively, and ate his food well; vomited two or three times, and was purged more than the other: he passed tape-worms, and the dejections were black.

"The dog that took ʒiij. was heavy, and apparently uncomfortable the whole day, but did not vomit at all. He was purged, and passed a very long tape-worm; dejections also black. Although he looked somewhat heavy before he took the calomel, and was apparently dull and uncomfortable during the day on which the calomel was administered, he improved very much in his appearance on the following day, and was very lively.

"At ten o'clock, A. M. on the 2d December, twenty-four hours from the time at which the calomel was taken, the three dogs were hanged; and as the largest dose was given with a view of ascertaining the worst effects of this preparation, I first examined the dog that took it, five minutes after he was dead.

"The veins were beautifully injected, the liver healthy, and the gall-bladder *full of bile.*

"The external coat of the stomach was of a pale colour, and seemed to be rather thickened.

"The small intestines had a peculiarly thickened feel, very similar to what is observed in cases of cholera; but I am not quite sure whether this thickened state is not natural to the healthy intestine of the dog.

"The stomach was laid open: its internal surface was considerably corrugated, and of a dusky red colour, but possessing neither the appearance of high arterial action, or of venous congestion. The corrugations were in a longitudinal, and not in a circular direction.

"The small intestines were laid open: their internal surface was loaded with thick, tenacious, cream-coloured matter, such as is generally found in the intestines of those who die of cholera. It appeared that the calomel, in this instance, had no other effect upon the dog than that of diminishing the vascularity of the stomach, as it did not seem to have mixed at all with the secreted matter of the intestines, or to have acted upon the gall-bladder. Probably the time was not sufficient for the purpose.

"The dog which was next opened, was that which took ʒij. of calomel.

"The appearance of the stomach, both externally and internally, was infinitely more vascular than observed in the preceding experiment. The corrugations were, however, precisely of the same nature as those already described, and the venous system was beautifully injected; but there was a very considerable flow of bile in this dog, and the contents of the duodenum were more fluid, and less tenacious.

"The dog who took ʒj. was last opened, and in him also the venous system was highly injected; but we were surprised to see a much higher degree of vascularity in the stomach of this dog, particularly at the internal surface, than in either of the two others.

"The bile, too, had flowed freely into the duodenum, and the contents of the bowel were highly coloured with bile, and not at all tenacious. The corrugations were of the same character, viz. longitudinal.

"Observing that the vascularity of the stomach was greatest in the dog which took the smallest quantity of calomel, I procured a healthy dog, and without giving him any of this preparation, had him hanged, and examined five minutes after he was dead, in order to see the natural state of the stomach—at least unoperated upon, and unchanged by any medicine. I was greatly surprised to find that the stomach of this dog was infinitely more vascular than that of either of the three dogs already examined, and was in what I really would have considered a high state of inflammation. The corrugations were circular, and more or less vascularity extended throughout the alimentary canal, which was covered with a glairy transparent mucus." 393.

In order to ascertain the correctness of the foregoing particulars, two other dogs were kept locked up and fed on rice and sheep's head for two or three days, and then three drachms of calomel were given to each. They both became sick, and vomited, but seemed to suffer no other inconvenience, for they ate well, and appeared in good spirits. On the 2d day after this, one of the dogs was killed by hanging, (the other lived and thrived well) and the stomach, externally, was found pale, with some large blood vessels spread over it. It was much distended with rice. When the rice was cleared out, the internal surface exhibited " a beautifully corrugated appearance throughout, with a peculiarly pale, pink blush—but nothing like excitement or vascularity was evident." The whole surface of the duodenum was covered with healthy bile, but without any vascularity or viscid secretion. The inner surface of the colon was in a state of high arterial vascularity, as was likewise the rectum.

" From comparing the state of this dog's stomach with those which I first examined, it seems that the first effect of calomel, in large doses, is not only to diminish vascularity, but also to produce a peculiar action of the fibres of the stomach, and that this organ requires a certain period to elapse before it can resume its natural function.

" The appearances, on examination of another dog which had not taken calomel, were as follow :—

" The stomach was found corrugated transversely ; it was in a much higher degree of vascularity than in the case of the stomach of the dog which had taken calomel, and this vascularity extended throughout the duodenum ; but the lower part of the intestinal canal was less vascular than the stomach ; its surface was covered with a viscid, glairy, and transparent secretion, adhering to and spread over the whole intestine.

" The corrugations of the colon had precisely the same appearance as those in the stomach—they were transverse. The rectum was not at all vascular, and the corrugations in its inner coat were in the longitudinal direction.

" The accompanying drawings, taken from the subjects at the time, will shew the actual state of the internal surface of the stomach and intestinal canal more clearly than any description ; and from them it will appear that calomel, even in these excessive doses, has the effect of diminishing vascular action, rather than of exciting it, which will account, in some degree, for the scruple doses of calomel at once allaying irritability of stomach and vomiting—a circumstance I have witnessed with astonishment, and for which I never could account till now." 397.

The foregoing experiments were followed up by others, the results being always the same. Our author is, therefore, led to the following inferences, (the first of which is corroborated by Dr. Yellowly's paper on the vascular appearance of the human stomach)—" that the natural and healthy state of the stomach and intestines is high vascularity ; and that the operation of calomel in large doses is directly the reverse of inflammatory."

For the correctness of the last part of this quotation we cannot pretend to answer ; but for the truth of the first part we can vouch. It requires not, indeed, the aid of experiments or vivisections to convince us that all the mucous surfaces, and especially that of the stomach and intestines, are red and highly vascular in a state of health. Look at the mouth and fauces—

look at the internal surface of the rectum when extruded, as is sometimes the case in children after straining or crying—and you will be convinced that the same or a still greater degree of redness and vascularity must exist in the other portions of the same tube. During life the capillaries of the interior surfaces are full. In death the blood forsakes those vessels, as well as the vessels of the exterior surface, and a death-like pallor pervades them. In dissection, therefore, we do not see the natural state of these parts. But as death blanches the interior as well as the exterior, it is fair, we think, to conclude that redness and vascularity found in the alimentary canal after a certain number of hours, are indicative of either irritation or inflammation there previous to the cessation of life. It is difficult, however, to conceive how a large dose of calomel can have the effect of diminishing the vascularity of the mucous surfaces, which the experiments of Mr. Annesley seem to prove. We can aver, nevertheless, from personal feeling and observation, that the said medicine has the effect of lessening irritation there. It is possible that it diminishes *sensibility*, in the same way that the nitrate of silver lessens the sensibility and irritability of sores, and thus it may diminish vascularity.*

*Influence of Calomel on the Hepatic and Intestinal Secretions.*—This subject deserves further investigation. Mr. Annesley has made experiments on the dead subject, and has observed "that the tenacious secretion which is frequently found covering the mucous coat, is completely changed by the admixture of a small quantity of calomel with it, *in situ*. This secretion assumes a dark grey colour, becomes more fluid, much less tenacious, and is easily detached from the mucous surface."

"The dark grey appearance communicated by the calomel to the secretion, covering the mucous coat of the intestines, is only remarked when there is no admixture of bile; and it is remarkable, that this appearance is precisely the same with that which the alvine dejections assume after the administration of a full dose of calomel, in the acute diseases of India, and before the biliary secretions appear in the stools; thus shewing the effect of the calomel upon the mucous secretions, in conjunction with its purgative operation, before it has succeeded in procuring the flow of bile either from the gall-bladder, or immediately from the liver itself." 399.

In thus separating the tenacious matter from the mucous coat of the intestines, Mr. Annesley thinks it probable that it may

* We know that epilepsy very often depends on irritation in the intestinal canal. Is it by lessening irritability there that lunar caustic diminishes the number and violence of epileptic seizures?

remove obstructions, from the bile-duct opening into the duodenum, and thus effect a discharge of bile into the intestine, which had been mechanically impeded in its progress to its destination. But further:—The mucous surface of the intestines having been cleared by the *chemical* operation of the calomel, and the purgative power of the cathartic draught, the bowels become more sensible to the succeeding doses, the influence of which may be propagated thence along the canals of the ducts to the gall-bladder and to the liver itself. This, we imagine, is the more philosophical explanation of the two.

"When a loaded state of the gall-bladder is inferred, from the presence of weight and oppression at the epigastrium, with a sense of coldness at the stomach, and various dyspeptic symptoms, then the purgative operation of calomel will succeed in procuring the discharge of the bile, unless there be a total obliteration of the canal through which it has to pass; and this and other purgatives ought to be employed until dark or dark green motions are procured—a colour which indicates that the flow of bile has taken place; as shewn by several trials I have made of the appearance which the matters contained in the intestines of recently dead subjects assume, when their tenacious mucous secretion, and a small quantity of calomel, are mixed *in situ*, and the bile lodged in the gall-bladder is poured upon the whole. In the first instance, as already pointed out, the admixture of calomel with this tenacious, mucous secretion and the feculent matter, produces a dark grey and pultaceous compound, similar to the first dejections proceeding from the exhibition of calomel before the flow of bile has taken place; in the second instance, a dark green and more fluid compound is formed, similar to the character of the motions, when the biliary evacuation is occasioned by the use of this remedy.

"When, therefore, we see the change from dark grey—the colour which calomel alone gives the mucous secretion—to dark green, we may rest satisfied that the ducts are emulged, and that the calomel and cystic bile are acting conjointly upon the bowels. Hence, the propriety of continuing this remedy till healthy action be produced, will appear evident." 403.

Mr. Annesley considers the viscid secretion abovementioned to be morbidly increased during many acute diseases, and particularly those which prevail in India. This, he thinks, may give to the intestines a thickened appearance, when viewed externally; while, upon laying them open, an enormous quantity of the tenacious matter becomes evident. This is especially the case where purgation has been neglected in the course of the disease. Hence the utility of calomel in preparing the offending matter to be acted on by other purgatives, rendering it more fluid, and less adhesive to the coats of the intestines. Add to this the effect of calomel in rendering the mucous surface of the stomach less vascular, if the experiments made are to be admitted as conclusive.

In fever, dysentery, and liver complaints then, our author has been in the habit of giving at bed time, 20 grains of calomel, with one or two grains of opium, and sometimes without this last, following it up next morning by a smart purgative draught. This practice he repeats daily until the excretions assume a healthy state. A tonic laxative is then exhibited till the bowels resume their natural functions. Salivation is always avoided if possible.

*Calomel in Fevers.* In these the medicine is given with three distinct views—to diminish the irritability of the stomach—to correct and promote the discharge of the secretions on the internal surface of the stomach and intestines, as well as of the associated glandular organs—and, thirdly, (which is included in the second indication) to procure "increased action of the great secreting organs."

Our author goes on to the mode of administering the remedy in the different *types* of fever, but these modifications we deem it unnecessary to dwell upon here.

*Acute Hepatitis.* Next to general and local depletion, Mr. Annesley ranks calomel. Here, as elsewhere, he gives it in large doses, and with the views which have been already pourtrayed. The dread of a sore mouth seems to haunt his imagination day and night. While he admits that calomel lessens vascularity in the stomach, and controls inflammation in the liver, yet he conceives, on what grounds we know not, that the moment the gums are sore, all its salutary operations are reversed—the energies and "vital resistance" of the system are impaired—and, in short, the local inflammation, excitement, and irritative action in the liver is kept up, instead of being still farther reduced. Knowing as we do, from personal observation and feeling, that all this is purely imaginary, we confess that our confidence in Mr. Annesley's opinions is thereby somewhat lessened. In the acute hepatitis of India, we know, from woeful experience, that the liver and the life are hardly ever safe, till some degree of soreness takes place in the mouth, and on the truth of this statement, as ascertained by the experience of those who make a fair trial of the two methods, we stake the issue of the difference between Mr. Annesley and ourselves. We would, and always have put the means recommended by Mr. Annesley into execution, but we advise the carrying the measures to a moderate constitutional affection, when the local symptoms will be found to give way with accelerated speed.

*Chronic Hepatitis.* In the simple, and also the more complicated forms of chronic hepatitis, mercury, says Mr. A. may be given as a purgative—but never as a sialagogue. The old precaution is continually repeated—take care not to make the mouth sore! We have not the smallest doubt but that the peculiar practice of our author gave him numerous opportunities of observing the following picture, so little creditable to the power of medicine.

"The derangement now described may be neglected, *or it may be partially removed,* in which case it generally returns. In either instance it will terminate, in a longer or shorter time, according to circumstances, in more serious disorder. It will give rise to vascular action, of a sub-acute kind, in the substance of the liver, which, whilst tending to overcome the obstruction previously existing, also gives rise to the effusion of lymph in the structure of the part where such reaction supervenes. Thus, enlargements of parts, or of the whole of the liver, take place; or the formation of tubercles and scirrhous hardness is the result, even although the patient may not have been the subject of previous acute disease of this viscus. It ought, however, to be remarked, that this, as well as the other chronic derangements of the biliary organs, are often the result of a previous attack or attacks of acute hepatitis; but whether occurring as the sequelæ of the acute form of the disease, or as the primary disorder, either in a patient who has enjoyed previous good health, or in one who has suffered under some other disorder, as intermittent, or remittent fever, &c. the symptoms and the treatment will be nearly the same in most of their important constituents.

"The form of chronic disorder now under consideration is indicated by the presence of the greater number of the symptoms already detailed, with the addition of a dull pain under the blade or top of the right shoulder, with uneasiness, and occasional pain and fulness in the region of the liver, or at the epigastrium, with a white or foul tongue, dry harsh state of the skin, occasional slight paroxysms of fever, &c. This form of disorder requires the exhibition of calomel in the manner already pointed out, in conjunction with local depletions, blistering on the region of the liver, warm poulticing, and the nitro-muriatic acid bath. In this form of disorder, the alternate use of large doses of calomel, and the cathartic draught, is required for a longer period, and the subsequent employment of aperients and laxatives should also be longer persisted in. More caution is requisite in resorting to the exhibition of tonics, and these should never be prescribed uncombined with aperients or laxatives." 430.

*Acute Dysentery.* It is quite needless to pursue Mr. Annesley's work any farther. The scruple of calomel at night, with the black draught in the morning, is the grand panacea for every ill.

We have now done our duty in making our readers acquainted with our author's ideas. They are rather too exclusive, we fear, to be always correct. We should have thought Mr. A. had lived long enough in the world to have known this; but we see that this is not the case. We leave his opinions in the hands of his oriental brethren, who are well able to appreciate them.

We now proceed to give some account of a paper written by an American physician, and published a month or two before Mr. Annesley's work appeared.

### *An Essay on Syphilis.* By S. A. CARTWRIGHT, M.D.

[American Medical Recorder, July, 1825.]

Accident more frequently leads to the discovery of remedial agents than design. Dr. Cartwright was called on, in the autumn of 1822, to treat a gentleman for syphilis, of about a month's standing—chancres and buboes. Dr. C. commenced his usual plan of two grains of calomel, guarded with opium, per diem. In a day or two the patient was attacked with the bilious fever then epidemic in the country—Mississipi. His stomach was very irritable, and Dr. C. gave him daily two doses, consisting each of a scruple of calomel, working them off, if they did not work themselves off, with purgative enemata. This course was pursued for a week, when it was suspended, as the fever was gone, and he complained of tenderness in the gums. The original complaint was now attended to, but the chancres were healed and the buboes were dispersed. The patient used no more medicine, and twelve months afterwards he had no return of syphilitic symptoms. This case led Dr. Cartwright to try a nearly similar plan in many others, and all with the same effect. The plan will be seen in the following extract.

"Did I deem it necessary, I could relate numerous other cases of the worst kind, wherein there were buboes, ulcers in the throat, caries of the bones and nodes, which have yielded nearly as soon to the above plan, as a recent infection would have done to the common mode of treatment. As it respects recent cases, they have been found by an experience of more than two years practice in syphilis, to yield almost as soon to twenty or thirty grains of calomel, given every, or every other day, as a common cold in good constitutions yields to the native powers of the system. I never prescribe the calomel with a view to produce ptyalism; to guard against such an event, I always direct an injection to be given, or some mild purgative taken, in twelve or sixteen hours after the calomel, if it does not operate. And in the event of its operating too much, I direct a little laudanum, so as to limit its action on the bowels to two or three evacuations, unless they be of a deep green or black colour. As soon as a copperish taste is perceived in the mouth, or the least tenderness of the gums, or soreness of the teeth, I order its immediate suspension, until these symptoms have disappeared; when it should be resumed with caution. The preparation I generally use is 20 grs. of calomel and 4 of rhubarb, given at bed time. Generally, by the time three or four doses have been taken, the breath will begin to have a mercurial odour, a copperish taste will be perceived in the mouth, or the gums will feel tender. About this time, or even before it, the venereal symptoms begin to disappear, and in a few days more, the chancres entirely heal. I generally recommend, after the healing of the chancres, a dose or two more, to ensure the complete elimination of the disease. I have rarely found more than twelve or sixteen pills, each 10 grs. calomel, and 2 rhei, (two pills every night) necessary for the cure of a recent infection. On asking an intelligent physician of this place, (Dr. Chew,) if he did not often find great difficulty in bringing the system of a robust venereal patient under the specific influence of mercury by the use of corrosive sublimate, the blue pill, small doses of calomel, or frictions with mercurial ointment, he informed me, that he had so often seen *no* effect at all from these remedies, or such *bad* effects, that he had made it a rule of practice to prepare the system for a mercurial course, by a large dose or two of calomel. My plan differs from his in this—that, after having wrought *a change* by efficient doses of calomel, instead of putting the patient on the old plan of treatment, I keep up *this change of state in the system* by an occasional dose of calomel, which soon cures the patient. He is generally able to attend to his common employment, to eat at the same table, to sleep in the same bed with his acquaintances, without its being discovered that he is taking any medicine at all, and, if the physician chooses, without the patient himself knowing that he is taking mercury. The only local application I use, is a soft rag spread with simple ointment, or ol. oliv. to be retained over the diseased parts, to prevent them from becoming irritated. Should they be much inflamed, soft poultices of slippery elm bark, until the inflammation is removed. The patient should be temperate in his diet, and should drink, if convenient, of a tea made of the wood of the root of the laurus sassafras. The physiological changes which I have observed to take place, in the systems of those whom I have so often put under the above plan of treatment, are 1st. an extraordinary excitement of the hepatic secretories, which is proven by two, three, or more copious, dark or green bilious evacuations; 2d. an extraordinary excitement of the exhalents of the skin, which is proven by the strong smell of the patient's linen, and the rapidity with which it becomes soiled; 3d. an increased excitement of the renal secretories, as manifested by an increased secretion of urine; 4th. the absorbent system receives new vigour, as is proven by the rapid resolution of buboes and other swellings. The patients, under the above course, are generally able to attend to their ordinary employments." 446.

Before proceeding farther, it may be proper to state that, several years ago, Mr. Cunningham, a naval surgeon, published some cases of syphilis which he cured in a nearly similar way, viz. by scruple doses of calomel, till the mouth became sore, when the chancres quickly healed, the whole period of cure occupying a much less space of time than in the ordinary way. Mr. Boyle, now surgeon of the Middlesex Infirmary in this metropolis, more recently published cases treated in a similar manner. The American plan, if equally efficient in securing against secondary symptoms, has certainly the advantage, as it does not produce ptyalism, or at least very little. The patient may also follow his usual avocations during the treatment. The plan is worthy of a trial.

The most remarkable circumstance in Dr. Cartwright's paper is a complete coincidence between himself and Mr. Annesley in this country (Dr. C. having a month or two the start of Mr. A. in point of publication) respecting the treatment of fever, dysentery, and several other complaints by means of scruple doses of calomel, given at such intervals as may prevent ptyalism, the object being to act on the bowels and on all the excretories, without running particularly to the mouth. A few short extracts will shew this coincidence.

"Those who have not used calomel extensively, would be apt to suppose, *à priori*, that large doses would produce hyper-catharsis, and debilitate the patient. But experience can best refute such suppositions, for it shows us that large doses of calomel operate much more mildly than smaller ones." 447.

"That large doses of calomel purge *less* than smaller doses of that medicine, although well known to the experienced practitioner, appears yet to be viewed as an insulated fact, not well understood. Nothing, however, will be more plain, if the phenomena the system presents, under the influence of large doses of calomel, be observed. These phenomena are an increased secretory action, not in one organ alone, but in all (or at least many) secretory organs of the system. Hence the secretions *of no one particular organ can be very profuse*, when *all* the organs are preternaturally excited. Smaller doses act more directly on one secretory organ—that organ is the *liver*. And hence the secretion of bile is very profuse, because no organ but the liver is preternaturally excited. The truth of this fact, the experience of every practitioner, who has used calomel in doses of from five to fifteen grs. will establish. Still smaller doses, if not sufficient to produce secretion, act on the system as a morbid irritant, and occasion mercurial fever, which nature removes by an increased secretion of the salivary glands." 448.

"In proof that large doses of calomel not only cure syphilis by producing an increased secretory action of many organs, but bilious fever, dysentery, &c. and that this general secretory action is incompatible with an immoderate secretion from any one organ, as the liver or salivary glands, in addition to the proofs already adduced, in which salivation, or an immoderate secretion of the salivary glands became diminished, when large doses of calomel had produced a secretory action in other organs, I beg leave to refer to the case of Dr. Johnson, as related by himself, in his Tropical Climates, under the head of Dysentery." 449.

"As to myself, I have, in my practice, seen worse effects from twenty grs. of calomel, divided into twenty parts, than I ever did, from twenty doses, each twenty grs. I could bring forward numberless facts, to prove the superiority of larger, over smaller doses, wherein, what is commonly called the specific effects of mercury were indicated, but I deem the following sufficient. I had a case of hydrothorax, which, I believed would end fatally. I called on my friend, (Dr. Isaac Butler, of Florence, Alabama,) to consult with me on the course which should be pursued. I told him, that I had been giving, without effect, calomel in small doses, combined with squills. He recommended scruple doses. On my expressing fears of bad effects from such doses, he asked me, if I would fear their effects if the case were bilious fever or dysentery? I hesitated no longer, but gave scruple doses repeatedly. The dormant secretory organs were aroused throughout the system. The kidnies, liver, and skin poured forth their specific fluids. The serous membrane of the thorax no longer bore the burden of maintaining the principal secretory action of the system, as it must have done, when the above important organs of secretion lay powerless. The loss of balance in the secretory action of the various organs, which constitutes the essence of dropsy, was restored, and health returned." 455.

The above will shew a very striking coincidence between two practitioners, removed from each other a distance equal to the diameter of this earth. As both brought forward their observations at nearly the same time, there can be no reason to give the priority to either.

